# Orthodontic and Facial Characteristics of Craniofacial Syndromic Children with Obstructive Sleep Apnea

**DOI:** 10.3390/diagnostics13132213

**Published:** 2023-06-29

**Authors:** Suliman Alsaeed, Nelly Huynh, David Wensley, Kevin Lee, Mona M. Hamoda, Evan Ayers, Kate Sutherland, Fernanda R. Almeida

**Affiliations:** 1 Preventive Dental Sciences Department, College of Dentistry, King Saud bin Abdulaziz University for Health Sciences, Riyadh 14611, Saudi Arabia; 2 King Abdullah International Medical Research Center, Riyadh 11481, Saudi Arabia; 3 Ministry of the National Guard—Health Affairs, Riyadh 11426, Saudi Arabia; 4 Faculty of Dental Medicine, Université de Montréal, Montreal, QC 2001, Canada; nelly.huynh@umontreal.ca; 5 Faculty of Medicine, University of British Columbia, Vancouver, BC 2312, Canada; dwensley@cw.bc.ca; 6 Faculty of Dentistry, University of British Columbia, Vancouver, BC 2199, Canada; kevinleedmd2011@gmail.com (K.L.); monamoda@mail.ubc.ca (M.M.H.); evan.ayers@gmail.com (E.A.); falmeida@dentistry.ubc.ca (F.R.A.); 7 Sleep Research Group, Charles Perkins Centre, Faculty of Medicine and Health, University of Sydney, Sydney, NSW 2006, Australia; kate.sutherland@sydney.edu.au; 8 Centre for Sleep Health and Research, Department of Respiratory and Sleep Medicine, Royal North Shore Hospital, Sydney, NSW 2065, Australia

**Keywords:** pediatric, syndromes, polysomnography, apnea–hypopnea index, diagnostic imaging

## Abstract

**Introduction:** Obstructive sleep apnea (OSA) is a disorder in which ventilation becomes disrupted due to a complete or partial upper airway obstruction Altered craniofacial morphology is one of the most important anatomical factors associated with obstructive sleep apnea (OSA). Studies have assessed craniofacial features in the non-syndromic pediatric population. The aim of this study was to analyze the orthodontic and facial characteristic of craniofacial syndromic children referred for polysomnography (PSG) and to assess the correlation with the apnea–hypopnea index (AHI). **Methods:** In the current cross-sectional study, consecutive syndromic patients referred for PSG were invited to participate. A systematic clinical examination including extra- and intra-oral orthodontic examination was performed by calibrated orthodontists. Standardized frontal and profile photographs with reference points were taken and analyzed using ImageJ^®^ software to study the craniofacial morphology. PSG data were analyzed for correlation with craniofacial features. STROBE guidelines were strictly adopted during the research presentation. **Results:** The sample included 52 syndromic patients (50% females, mean age 9.38 ± 3.36 years) diagnosed with 17 different syndromes, of which 24 patients had craniofacial photography analysis carried out. Most of the sample (40%) had severe OSA, while only 5.8% had no OSA. Down’s syndrome (DS) was the most common syndrome (40%) followed by Goldenhar syndrome (5%), Pierre Robin Sequence (5%), and other syndromes. The severity of AHI was significantly correlated with decreased midfacial height. increased thyromental angle and cervicomental angle, decreased mandibular angle, and decreased upper facial height. All patients with DS were diagnosed with OSA (57% severe OSA), and their ODI was significantly correlated with increased intercanthal distance. Obesity was not correlated to the severity of AHI for syndromic patients. **Conclusions:** Decreased midfacial height and obtuse thyromental angle were correlated with increased AHI for syndromic patients. Increased intercanthal distance of DS patients could be a major predictor of OSA severity. Obesity does not seem to play a major role in the severity of OSA for syndromic patients. Further studies with larger samples are necessary to confirm these findings.

## 1. Introduction

Obstructive sleep apnea (OSA) is a disorder in which ventilation becomes disrupted due to a complete or partial upper airway obstruction [1]. The gold standard diagnostic aid for adult or pediatric OSA is polysomnography (PSG) [2]. However, pediatric OSA is different from adult OSA in terms of risk factors, treatment options, and diagnostic criteria [3]. It is estimated that 1 to 6% of children experience OSA, and this percentage reaches 70–100% for children with craniofacial syndromes, making it a major risk factor [4].

Down’s syndrome, Goldenhar syndrome, Pierre Robin Sequence, and Curzon’s syndrome are some of the main craniofacial syndromes that are associated with OSA [5]. This association can be attributed to neuromotor deficits that impair the ability to maintain a patent upper airway and also due to altered craniofacial morphology as described in depth in Table 1 [5].

Despite the presence of radiation, lateral cephalometric radiographs and cone beam computed tomography (CBCT) are considered two of the most widely used imaging techniques in dentistry to assess craniofacial characteristics [6]. However, a new standardized facial photography technique has been developed to evaluate craniofacial characteristics, especially in OSA patients [7]. This technique is based on the analysis of two photographs, facial and profile views, followed by a precise tracing, utilizing specific landmarks that are pre-marked on the patient before taking the photos [7]. This technique was validated on both adult and pediatric patients [7,8]. A study utilizing this technique found that adult patients with OSA, after matching for body mass index (BMI) and gender, had significantly shorter *mandibular length*, smaller *mandibular-nasion angle*, smaller *anterior neck space area*, larger *mandibular width to length angle*, and larger *face width to mid-face depth angle* compared to those with no OSA [7]. In previous work by our group, we found that young children with OSA (4 to 11 years old) presented with significantly increased *lateral facial height* compared to those with no OSA, while older children (12–16 years old) had similar lateral facial height, but with significantly increased *eye width* and *total facial height* than those with no OSA [8]. A similar study, but on a wider age range (2–18 years old), found a difference in *facial convexity angles* and *upper to lower facial height ratio* among SDB groups, including controls [9]. These studies help the clinician to accurately specify the craniofacial characteristics that correlate the most with the severity of OSA [7,8,9]. However, there have been no studies on a consecutive sample of syndromic children investigated with standardized photo analysis, which could reveal some craniofacial features that contribute or correlate with the severity of OSA. To achieve this goal, we performed this study on craniofacial syndromic children, analyzed their dental and facial characteristics, and studied their correlation with PSG parameters.

**Table 1:** Summary of the most common craniofacial syndromes and their association with OSA.

SYNDROME	EXTRA-ORAL FEATURES		INTRA-ORAL FEATURES	ASSOCIATION WITH OSA
**Down’s syndrome** **1:700** **Genetics: Trisomy 21+ mutation in GATA1 gene [10]**	**Face** ▪ lat nasal bridge ▪ Small ears ▪ Epicanthic folds ▪ Up slanting palpebral fissures **Cranium** ▪ Flat cranial base (N-S-Ba angle)	**Maxilla** ▪ Hypoplastic ▪ Deficient development of length, depth, height but usually not width of palate ▪ High V-shaped palate	▪ Macroglossia ▪ Microdontia of primary and permanent dentition ▪ Clinical crowns may be conical, shorter and smaller than normal and roots shorter ▪ Hypotonic perioral musculature ▪ Descending angles of mouth ▪ Everted lower lip w. tongue protrusion ▪ Mouth breathing ▪ Xerostomia	**OSA prevalence [11,12,13,14,15]** Childhood: 50–100%, Adulthood: 100% **Predisposing factors—related to the syndrome:** Macroglossia, adenotonsillar hypertrophy, midface hypoplasia, other associated conditions such as obesity, hypothyroidism, hypotonia, and gastroesophageal reflux [16]
Goldenhar syndrome 1:3500 to 1:7000 Genetics: *MYT1* [17]	**Face** ▪ Hemifacial microsomia ▪ Anterior-posterior and vertical dimensions reduced on affected side ▪ Lateral facial cleft ▪ Macrostomia **Eyes:** ▪ Epibulbar dermoids ▪ Narrowing of palpebral fissures ▪ Vertical dislocation of orbit ▪ Microphthalmia/anopthalmia ▪ Upper eyelid colobomas	**Ears** ▪ Pre-auricular tags ▪ Deformities of pinna ▪ Microtia (small external ear)/anotia ▪ Hearing loss **Vertebral anomalies** ▪ Scoliosis ▪ Abnormal rib structure (missing or fused) ▪ Hemi vertebrae and cervical fusion	▪ Micrognathia ▪ Hypoplasia of maxilla and mandible ▪ High arched palate ▪ Delayed tooth eruption ▪ Supernumerary/missing teeth ▪ Enamel and dentin malformations ▪ Gingival hypertrophy ▪ CLP	**OSA prevalence** Up to 67% [1] **Predisposing factors related to the syndrome:** Maxillomandibular hypoplasia, glossoptosis, and abnormal control of breathing in patients with neurological compromise [18].
Cerebral Palsy 1 in 322 [19] Genetics: GAD1 on chromosome 2q31 [10]	***Depends on the type and severity:*** ▪ Impaired motor control ▪ Delayed motor development ▪ Spastic muscles ▪ Difficulty walking	▪ Drolling ▪ Difficulty swallowing ▪ Ataxia ▪ Seizures	▪ Tongue thrust ▪ Mouth breathing ▪ Oral mucosa hypersensitivity ▪ Delayed eruption of permanent teeth ▪ Enamel hypoplasia ▪ Increased risk of dental caries ▪ Dental erosion	**Risk of OSA:** 38–55% [20] **Predisposing factors—***varies between CP types:* Abnormal muscle tone, inability to change position on bed and as a side effect of some medication “for those with epilepsy” [21]
Pierre Robin Sequence syndrome 1: 8500–14,000 Genetics SOX9, COL2A1 [10]	**Cardiac:** Cor pulmonale (right side heart failure) **Ears:** **infections/hearing loss ear** **Mandible:** **Retrognathia**		**Triad of:** 1. Cleft palate (U-shaped) 2. Mandibular retrognathism—but will still grow 3. Glossoptosis ▪ Posterior displacement of the tongue ▪ Lack of support of the tongue musculature ▪ Airway obstruction ▪ Hypodontia	**OSA prevalence** 85–100% [22,23] **Predisposing factors related to the syndrome:** Mandibular retrognathia Mechanical collapse of the pharyngeal wall [24]
DiGeorge syndrome 1:4000–7000 Genetics:TBX-1 [10]	**CATCH 22** Cardiac defects (Tetralogy of Fallot) Abnormal facial features Thymic hypoplasia Cleft palate Hypocalcemia Craniosynostosis	**Eyes:** Ocular hypertelorism, hooding of upper lids, ptosis, tortuous retinal vessels. **Nose:** prominent nasal base, bulbous nasal dimples. **Ears:** over folded, microtic ears, pre auricular pits.	Enamel defects Hypomineralization Higher risk of dental decay	**OSA prevalence** 10.2% [25] **Predisposing factors related to the syndrome:** Micrognathia and VPL surgeries
Treacher–Collin syndrome 1:25,000, 50,000 Genetics: Loss of function in gene TCOF-1 located on chromosome 5 [10]	**Face** Hypoplastic Zygoma Depressed cheeks Narrow face **Mandible** Underdeveloped mandible with retrusive chin Hypoplasia of condylar and coronoid processes Steep mandibular plane Prominent antegonial notching	**Eyes** Downward slanting palpebral fissures 75% have coloboma (notch on outer eyelid) **Ears** Deformed or displaced pinnae Extra ear tags Ossicle defects or absence of EA canal causing conductive hearing loss	Class II Malocclusion Open bite High arched palate Cleft palate in 30% of cases	**OSA prevalence** 29–95% [26,27] **Predisposing factors related to the syndrome:** Narrowing of the upper airway due to mandibular retrognathia and choanal atresia [28].
Prader–Willi syndrome 1:10,000–25,000 Genetics mutation chromosome 15q11-q13 [10]	**Face** Narrow bifrontal diameter Round face **Eyes** Almond-shaped eyes,	**Others** Small hands and feet Rapid weight gain Hyperphagia Hypogonadism	Downturned corners of the mouth Dental caries Enamel defects Tooth wear	**OSA prevalence** 44% to 100% [29,30] **Predisposing factors related to the syndrome:** Increase in viscosity of secretions Craniofacial abnormalities causing small airways, Hypotonia leading to airway collapsibility [31]
Apert syndrome 1:65,000–160,000 Genetics FGFR2 on chromosome 10q26 [10]	**Cranium:** Acrobrachycephally (tower skull) Kleeblatt-schadel (cloverleaf) Frontal bossing and tall forehead **Eyes:** Ocular proptosis Hypertelorism Downward slanting palpebral fissures Vision loss	**Ears** Middle ear infection Conductive hearing loss **Maxilla** Midface deficient = class III **Limbs** Syndactyly of 2nd, 3rd, and 4th digits and toes, 1st and 5th may be separate or joined	V-shaped arches Upper crowding Anterior open bite with posterior crossbite 75% have cleft of SP or bifid uvula Trapezoidal shaped lips Increased gingival thickening may be associated with Delayed eruption of teeth Shovel shaped incisors	**OSA prevalence** 81% [31] **Predisposing factors related to the syndrome:** Decreased size of nasopharynx Narrowing of post choanae = respiratory distress Increased mouth breathing = open mouth appearance
Achondroplasia 1:15,000–40,000 Genetics: FGFR 3 [10]	**Body** Dwarfism Short limbs Short fingers and toes **Facial** Underdeveloped midface Flattened nasal bridge	**Ears:** Increased ear infections **Cranium:** Hydrocephalus Short post cranial base	Retrognathic maxilla Normal mandible Protrusive maxillary incisors Anterior open bite Posterior crossbite Anterior reversed overjet	**OSA prevalence** 75% [32] **Predisposing factors related to the syndrome:** Increased airway resistance secondary to adenotonsillar hypertrophy

## 2. Materials and Methods

Ethical approval for this study was obtained from the University of British Columbia Research Ethics Board and BC Children’s Hospital (ethical approval # H12-03285). In this cross-sectional study, a consecutive sample of all craniofacial syndromic children (4–16 years old) referred for overnight sleep study (polysomnography—PSG) at the Respirology department at British Columbia Children’s Hospital were invited to participate in the study. All patients reported symptoms of OSA such as snoring, witnessed gasping, unrefreshed sleep, or night sweat. All patients received information about the study 2 weeks prior to their PSG, once they arrived for the PSG, they were asked if they were willing to participate, and only patients and parents who signed the assent and consent forms were included in the study. We took all the photos and assessment the night prior to the PSG. We excluded those who had started treatment for sleep apnea or who had had orthodontic treatment or a non-diagnostic PSG. STROBE guidelines were strictly adopted during the research presentation, and we complied with all the protocols. 

Every patient underwent an overnight, in-lab, level one PSG by a licensed and trained respiratory technologist. The duration of each study was 8–10 h, and it included continuous video monitoring in addition to overnight monitoring of an electroencephalogram, electro-oculogram, electro-cardiogram, chin and anterior tibial electromyogram, nasal pressure transducer, oral thermistor, a snore sensor, respiratory inductive plethysmography, pulse oximetry, and end-tidal capnography. According to the American Academy of Sleep Medicine handbook, one of BCCH’s four sleep technicians scored the studies using the XLTEC (Oakville, ON, Canada) data gathering and processing system.

### 2.1. Orofacial Examination

After obtaining the consent form, a comprehensive orofacial examination was performed by three calibrated orthodontists (M.M.H., E.A. and K.L.) to assess the patients’ extra- and intra-oral features and recorded in a standardized data collection form [33]. The extra-oral evaluation assessed facial symmetry, lower facial height, facial profile (convex, straight, concave), maxilla and mandible position (retrognathic, normal, prognathic), and lip incompetency. The intra-oral exam assessed overbite, overjet, molar classifications (Class I, II, or III), crossbite, tongue size, palatal width, amount of crowding or spacing, tonsils size, and presence of mouth breathing. In order to reflect the level of severity of malocclusion, we used the index of orthodontic treatment need (IOTN) to identify those who would benefit from orthodontic treatment [34]. 

### 2.2. Craniofacial Photography

In accordance with previously validated guidelines, frontal and profile photographs of the head and neck were taken for the syndromic patients using a single-lens digital camera (L830 Nikon Corp., Tokyo, Japan) [7]. Prior to the photographs, specific anatomical landmarks were pre-identified on the subjects by palpation, and marked using small, rounded stickers of various colors. These landmarks were *right infra-orbital ridge*, *right gonion*, *sternal notch*, soft tissue *gnathion* and *menton*. In order to allow for digital calibration during analysis and prevent errors from differences of the distance between subject and camera, 3.0 cm washers were taped to the forehead for the frontal photo, and on the right cheek for the profile photo.

As prescribed in Lee et al., the landmark digitization was performed using ImageJ Software, version 1.5, NIH, Bethesda, MD [7]. These landmarks were located on the images, then transferred to an Excel spreadsheet as x, y, pixel coordinates to allow for analysis in the forms of linear, angular, area, and volume measurements. 

The classification of OSA was as follows; AHI of 0–1.99 was categorized as no OSA, AHI of 2 to 4.99 as mild, 5 to 9.99 as moderate, and AHI of 10 or more as severe [35]. The sample was divided by the body mass index percentiles (BMIP) for non-obese and obese patients.

### 2.3. Statistical Analysis

Using SPSS Software (version 23, Chicago, IL, USA), the Student’s *t*-test (normally distributed) and Mann–Whitney U-test (not normally distributed) were used to analyze variables from clinical examinations and photograph analysis respectively. To analyze categorical variables, the Pearson chi-square test and Fisher’s exact test were used. To study the relationship between measurement analysis and the severity of OSA, a Pearson correlation coefficient was used. Frontal and profile photographs from 15 randomly selected participants were re-digitized 3 weeks apart to assess intra-examiner reliability and presented with intraclass correlation (ICC) scores.

## 3. Results

A total of 52 craniofacial syndromic children were consecutively recruited. The sample included syndromic patients with 17 different types of syndromes. Down’s syndrome (DS) was the most common (*n* = 21, 40%) followed by Goldenhar syndrome (*n* = 4, 7.7%), Cerebral palsy (*n* = 4, 7.7%), Pierre Robin Sequence (PRS) (*n* = 3, 6%), Treacher–Collins syndrome (TCS) (*n* = 3, 6%), DiGeorge syndrome (*n* = 3, 6%), Prader–Willi syndrome (*n* = 3, 6%), and Ehlers–Danlos syndrome (EDS) (*n* = 2, 3.8%), For the other nine syndromes, there was one patient (*n* = 1) for each, including fetal alcohol syndrome, frontometaphyseal dysplasia, 15q13.3 microdeletion syndrome, neurofibromatosis syndrome (NF type I), Joubert syndrome, Dubowitz syndrome, Ohdo syndrome, Coffin–Siris syndrome, and cleft lip and palate.

**Figure 1:**
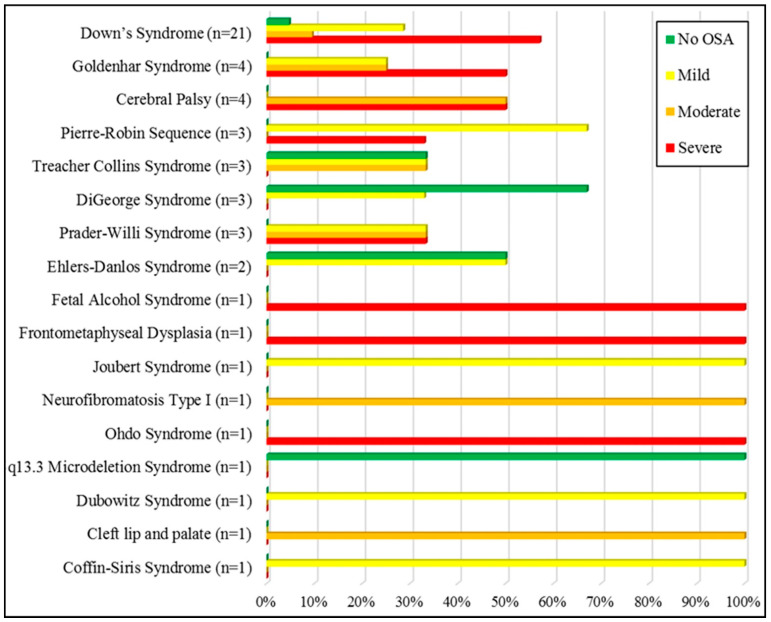
Distribution of OSA severity groups among the sample based on syndrome type.

Shown in Table 2 are the demographic distribution and PSG results for the total sample and OSA subgroups. The mean age of the sample was 9.38 ± 3.36. One-fourth of the patients were obese (27.7%), and 51% were female. No significant differences were found between the four OSA categories (no OSA, mild, moderate, and severe) in terms of age, gender, BMI, tonsils size, mouth breathing, size of tongue, arch shape, or palatal depth. Most of the sample (88.5%) were diagnosed with OSA, of whom, 46% were in the severe category. Only six patients (11.5%) were not diagnosed with OSA (two DiGeorge patients, one DS, one EDS, one TCS, and one 15q13.3 microdeletion syndrome). The distribution of OSA categories between the different syndromes is shown in Figure 1.

When DS patients (*n* = 21, mean age 10.02 ± 2.72) were analyzed separately, 95% had OSA, of whom, 60% were severe. Only 38% of them were found to be obese, with the majority (57%) being males. No significant differences were found between DS patients in terms of age, gender, or obesity that contributed to, or correlated with the severity of OSA.

## 4. Craniofacial Measurements

The intra-examiner reliability on landmark identification and craniofacial measurements varied between ICC of 0.76 for neck landmarks (moderate to good reliability) and 0.98 for soft tissue menton and infra-orbital notch (excellent reliability), with an average of 0.96 for all landmarks. 

**Figure 2:**
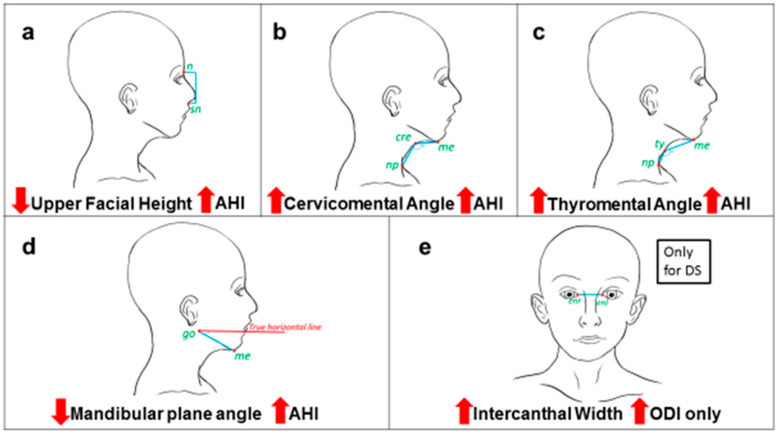
Illustration of the craniofacial measurements that were found to be correlated with OSA severity. n: nasion, sn: sub-nasion, go: gonion, me: menton, cer: cervical point, ty: thyroid, np: neck point, enr: endocanthion right, enl: endocanthion left. (**a**) decreased upper facial height was correlated with increase in AHI, (**b**) increased cervicomental angle was correlated with increase in AHI, (**c**) increased thyromental angle was correlated with increase in AHI, (**d**) decreased mandibular plane angle was correlated with increase in AHI, (**e**) increased intercanthal width was correlated with increase in ODI for Down’s syndrome patients only.

A total of 24 patients were accepted to participate in the photography section and fulfilled the criteria of acceptable record quality that enabled this analysis. The cases included DS patients (*n* = 13), Goldenhar syndrome (*n* = 3), Pierre Robin Sequence (*n* = 3), DiGeorge syndrome (*n* = 2), Prader–Willi syndrome (*n* = 1), frontometaphyseal dysplasia (*n* = 1), and Joubert syndrome (*n* = 1). Despite the different syndromic groups, most photographic measures showed similar patterns and the same direction of correlations. The increase in the severity of OSA of the included patients (*n* = 24), measured by AHI and oxygen desaturation index (ODI), was correlated with *increased thyromental angle* (r = +0.467, *p* = 0.021), *increased cervicomental angle* (r = +0.439, *p* = 0.032), *decreased mandibular angle* (r = −0.528, *p* = 0.008), *decreased upper facial height* (r = −0.572, *p* = 0.004) (see Figure 2). These measurements were also correlated with increased ODI as described in Table 3. After excluding obese patients (*n* = 6) from the analysis, these measurements continued to be correlated with increased AHI and ODI. When we evaluated only the DS patients (*n* = 13), an increase in *intercanthal distance* was significantly correlated with an increase in ODI (r = +0.577, *p* = 0.039), and the correlation remained significant even after correcting for age and BMI.

Seven craniofacial measurements were significantly different between obese and non-obese patients (Table 4). These included *cervicomental angle*, *total* and *lower facial height*, *face width*, *mandibular width*, *neck width*, and *neck depth* (Table 4). 

## 5. Dental Characteristics

Dental occlusion was Class III in 40% of the cases, followed by Class II malocclusion (38%), while 60% of the patients had edge-to-edge occlusion or negative overjet and 43% had posterior crossbite. Narrow palate was found in nearly 60% of the patients. Almost all DS (95%) were mouth breather and 67% had one or more oral habits (nail biting, thumb sucking, lip biting, or bruxism). Macroglossia was found in 81% of DS patients. Fifty two percent of the DS children had retrognathic maxilla. None of the dental findings were associated or correlated with the severity of OSA for the syndromic children (*n* = 52) or for DS children (*n* = 21). The main findings of the dental examination for each syndrome are presented in Table 5.

## 6. Discussion

This study was conducted to analyze the orthodontic and facial characteristic of craniofacial syndromic children referred for polysomnography (PSG) and to assess the correlation with the apnea–hypopnea index (AHI) and also to identify if the known OSA risk factors in healthy children play a role in the severity of OSA in syndromic patients. The findings from the photography analysis showed that specific craniofacial measurements such as *decreased upper facial height*, *decreased mandibular plane angle*, *increased thyromental angle, and increased cervicomental angle* were positively correlated with AHI and ODI, even after correcting for obesity and age. However, the level of correlation was moderate, and at most, it explains only 33% of the increase in OSA severity. Obesity, age, gender, tonsil size, and common craniofacial characteristics often thought to be related to OSA, such as retrusive mandible, narrow palate, increased palatal height, and mouth breathing were not found to be associated with increased severity of OSA in syndromic children. This might reflect that the causality of OSA in syndromic children is different from OSA in healthy ones [8,9]. Moreover, three calibrated orthodontists evaluated the orofacial characteristics of the included patients, and for the first time we described the main dental and orthodontic findings for syndromes like 15q13.3 microdeletion syndrome, Ohdo syndrome, Joubert syndrome, and Coffin–Siris syndrome. 

A portion of the results of this study was based on software analysis of two frontal and profile photographs, using a standardized process that helps to assess the craniofacial measurements in three planes [7]. Although the use of this techniques is increasing in the literature, it is important to interpret the results of the linear and area measurements with caution for samples with different obesity and age ranges [7,8]. To address this point, the data for this study were analyzed several times, with and without obese patients, with and without older patients (12–16 years old), to avoid the effect of growth in interpreting the results. This helped us to draw robust conclusions in regard to the effect of craniofacial measurements on the severity of OSA, which was found to be correlated with *decreased upper facial height*, *decreased mandibular plane angle*, *increased thyromental angle, and increased cervicomental angle.*

When we compared our results to previous studies on non-syndromic OSA children, we found that the craniofacial measurements which correlate with an increase in AHI or ODI in syndromic children were different from what was found in non-syndromic children, except for the *cervicomental angle* [8,9]. For example, it is known that a steep mandibular plane is more prevalent in non-syndromic OSA children [36,37]. However, on syndromic children, we found a significantly negative correlation between increased mandibular plane and AHI (*p* < 0.01), even after excluding obese patients. The reason we excluded obese patients on this analysis was due to the potential effect of excess neck fat that might results in inaccurate landmark identification, and subsequently inaccurate measurements. 

Previously our research group applied the same methodology on non-syndromic children and found that AHI was positively correlated with the increase in *cervicomental angle*, *mandibular width*, *eye width*, and *cricomental distance* [8]. A recent study on OSA children also found that increased *cervicomental angle* and decreased *upper to lower facial height ratio* were significantly correlated with the increase in AHI [9]. For adults, Lee et al. found that AHI was positively correlated with *neck depth*, *neck perimeter*, *face width*, *mandibular width*, and *mandibular width-length angle*, but only two of these measurements remained correlated with AHI after controlling for BMI and gender, which are *face width* and *mandibular width* [33]. None of the previously described measurements, except for increased *cervicomental angle*, were found to be correlated with AHI or ODI in our study, which reflects that the predisposing anatomical factors for OSA in non-syndromic children or adults might not be applicable to syndromic patients.

One of the main syndromes analyzed in this study was Down’s syndrome. Due to its high prevalence and common association with OSA, the American Academy of Pediatrics have recommended that DS children undergo a PSG by the age of 4 years, aiming to help clinicians to provide an early treatment, and ultimately, improved prognosis [16,38]. In addition, in terms of diagnosis, the use of oximetry for DS was found to have poor sensitivity [39]. Further techniques have been suggested in the literature; for example Skotko et al. proposed a model that can predict DS patients who are unlikely to have moderate or severe OSA, and thus might not require a PSG [40]. The model had a high negative predictive value for moderate-severe cases (90%) and less for mild cases (73%) [40]. Our study findings in regard to the craniofacial measurements that correlated with AHI or ODI, especially increased *intercanthal distance*, could be implemented in a model that helps to identify those with potentially moderate to severe OSA.

In terms of dentofacial characteristics, no correlations were found in this study between dental abnormalities and OSA severity for syndromic children or DS children. However, previous studies found that syndromic children with OSA have greater prevalence of retrognathic mandible, dolichocephalic face type, posterior crossbite, and narrow palate compared to non-syndromic OSA children [33]. For DS patients, a previous work found that the dentofacial features of DS with OSA were not significantly different from patients with DS but without OSA, except for a deeper palatal vault in the DS-OSA group [41]. Interestingly, the only dentofacial characteristics that were found to correlate with an increase in AHI for DS was an increase in upper inter-canine width [41]. This could be due to the effect of tongue pressure on a narrow maxilla that can cause flaring out of the upper canines and so, an increase in inter-canine width. 

This study is not without limitations. Despite our efforts to collect consecutive patients, we only achieved a small sample of syndromic non-OSA patients (6 patients) which limits our ability to accurately compare syndromic patients with and without OSA. However, we identified for the first time new measurements that correlated with an increase in AHI in syndromic children. As we evaluated only consecutive syndromic patients who agreed to participate during the 2 years of the study, it was challenging to collect more than 52 craniofacial patients with complete data. Our pediatric sleep laboratory is small, and the dentist researcher could only be there for 2 nights/week, limiting the total number of patients. Despite this, this is the first study to assess with standardized photography the craniofacial anatomy of syndromic children and may lead to future focused research. In the interpretation of the results, care is needed as there were multiple comparisons for the correlation with AHI and ODI to craniofacial variables, but due to the small sample we did not correct for it.

Although the use of photography analysis does not necessarily reflect the skeletal relationship and can be affected by ethnic variations [9], it has been shown to have value in identifying the features associated with OSA severity in children or adults [7,8,9]. It is unlikely that our findings were affected by ethnic differences as most of our sample was comprised of Caucasians. However, there are other factors that can affect the severity of OSA that were not addressed in this study including the severity of the craniofacial abnormality of the syndrome, night to night variability of OSA [42], or other medical conditions associated with OSA. 

## 7. Conclusions

1. Specific craniofacial measurements were found to be correlated with the severity of OSA for craniofacial syndromic patients. Decreased upper facial height and mandibular plane angle were correlated with an increase in AHI and ODI, while increased cervicomental and thyromental angles were correlated with increase in AHI and ODI.
2. In this study, for Down’s syndrome patients, an increase in intercanthal width was correlated with an increase in ODI.
3. Considering the limitation of this study, dental characteristics of syndromic children with OSA do not seem to be different from non-OSA syndromic children. However, studies on a larger sample of non-OSA syndromic children are needed.
4. Although some craniofacial measurements were moderately correlated with AHI, this does not support craniofacial prediction models for OSA in syndromic children at this point, and larger numbers would be needed to establish clinical utility.

## Figures and Tables

**Table 2:** Demographic distribution and PSG results of syndromic children (*n* = 52).

	All (*n* = 52)	Non-OSA *(n* = 6)	Mild OSA (*n* = 16)	Moderate OSA (*n* = 9)	Severe OSA (*n* = 21)
	Mean ± SD	Mean ± SD	Mean ± SD	Mean ± SD	Mean ± SD
Age	9.38 ± 3.36	11.00 ± 2.83	8.00 ± 3.06	7.44 ± 2.69	10.71 ± 3.32
Males (%)	25/51 (49.0%)	3/6 (50%)	8/16 (50%)	3/8 (37.5%)	11/21 (52.4%)
Caucasians (%)	36/52 (69.2%)	6/6 (100%)	9/16 (56.3%)	7/9 (77.8%)	14/21 (66.7%)
BMI	20.11 ± 6.78	20.04 ± 2.63	19.41 ± 7.47	19.06 ± 6.66	21.08 ± 7.43
Obese	13/47 (27.7%)	0/6 (0%)	5/15 (33.3%)	2/7 (28.6%)	6/19 (31.6%)
Tonsils ≥3	16/52 (30.8%)	3/6 (50%)	2/16 (12.5%)	4/9 (44.4%)	7/21 (33.3%)
Mouth Breather	44/49 (89.8%)	6/6 (100%)	13/15 (86.7%)	8/8 (100%)	17/20 (85%)
Total Sleep Time (min)	375.49 ± 65.85	401.08 ± 42.95	346.86 ± 72.51	413.20 ± 35.94	373.82 ± 68.13
Sleep Efficiency (%)	81.15 ± 11.66	84.78 ± 7.76	76.65 ± 11.83	87.27 ± 8.70	80.92 ± 12.62
Sleep Latency (min)	31.58 ± 23.39	31.75 ± 14.22	39.03 ± 30.27	21.49 ± 16.00	30.19 ± 21.49
Mean O_2_ Saturation (%)	95.11 ± 2.09	96.00 ± 2.25	95.41 ± 1.94	95.37 ± 2.41	94.52 ± 2.02
Mean Heart Rate (bpm)	83.14 ± 13.92	77.42 ± 17.03	81.31 ± 11.98	85.76 ± 13.22	85.16 ± 14.96
AHI Index (events/h)	13.04 ± 18.36	1.02 ± 0.66	3.14 ± 0.70	6.86 ± 1.32	26.67 ± 22.89

**Table 3:** Relationships between craniofacial measurements and PSG data on 24 syndromic patients.

Measurements	Correlation (r)	
	AHI (r)	ODI (r)
Total face height (cm)	−0.336	−3.11
Upper face height (cm)	−0.572 **	−0.536 *
Lower face height (cm)	−0.193	−0.153
Upper to lower facial height ratio	−0.357	−0.347
Lateral facial height (cm)	−0.065	−0.137
Face width (cm)	0.110	0.123
Eye width (cm)	−0.107	−0.060
Mandibular length (cm)	0.102	0.053
Mandibular width (cm)	0.165	0.210
Neck width (cm)	0.107	0.172
Neck depth (cm)	0.146	0.189
Mid-face depth (cm)	−0.010	−0.059
Maxillary-mandibular relationship angle (°)	−0.164	−0.296
Mandibular width-length angle (°)	−0.037	−0.041
Mandibular plane angle (°)	−0.528 **	−0.487 *
Face width-midface depth angle (°)	−0.029	−0.005
Thyromental angle (°)	0.467 *	0.414 *
Cricomental distance (cm)	−0.053	−0.221
Cricomandibular distance (cm)	−0.036	−0.082
Cervicomental angle (°)	0.439 *	0.431 *

* *p* < 0.05, ** *p* < 0.01.

**Table 4:** Effect of obesity on AHI and craniofacial morphology on 22 patients (2 of the 24 patients had missing data on BMI).

Measurements	Non-Obese (*n* = 16)	Obese (*n* = 6)
AHI (events/h)	19.5	13.1
Total face height (cm)	11.5	12.8 *
Upper face height (cm)	4.2	4.3
Lower face height (cm)	5.3	6.3 *
Lateral facial height (cm)	9.2	9.6
Face width (cm)	12.9	14.4 *
Eye width (cm)	2.6	2.9
Mandibular length (cm)	6.6	7.3
Mandibular width (cm)	10.9	12.6 *
Neck width (cm)	9.4	11.5 *
Neck depth (cm)	9.9	12.8 *
Mid-face depth (cm)	9.2	10.5
Maxillary-mandibular relationship angle (°)	7.7	6.9
Mandibular width-length angle (°)	79.1	80.7
Mandibular plane angle (°)	24.2	19.6
Face width-midface depth angle (°)	64.4	66.0
Thyromental angle (°)	132.6	145.0
Cricomental distance (cm)	5.6	5.5
Cricomandibular distance (cm)	5.1	8.8
Cervicomental angle (°)	129.8	149.7 *

* *p* < 0.05.

**Table 5:** Description of the most common findings based on syndrome type.

	Facial Profile	Increased Lower Facial Height	Mouth Breather	Retrognathic Maxilla	Retrognathic Mandible	Class II Malocclusion	Facial Asymmetry	Narrow Palate	Macroglossia	Crowding/Spacing	Lip Incompetency	IOTN Score	Others
**Down’s Syndrome** **(*n* = 21)**	Straight: 62% Concave: 33% Convex: 5%	70%	95%	52%	5%	5%	33%	57%	81%	Crowding: 67% Spacing: 29%	52%	6.3 ± 2.7	Anterior cross-bite: 48% Posterior cross-bite: 43%
**Goldenhar Syndrome (*n* = 4)**	Convex: 100%	100%	50%	No	100%	75%	100%	50%	75%	Crowding: 75%	No	10	Posterior cross-bite: 100%
**Cerebral Palsy** **(*n* = 4)**	Concave: 75% Convex: 25%	50%	100%	No	50%	50%	50%	50%	75%	Crowding: 75% Spacing: 25%	75%	9.5 ± 0.7	Anterior open-bite: 75% Anterior cross-bite: 25% Posterior cross-bite: 25%
**Pierre Robin Sequence (*n* = 3)**	Straight: 33% Convex: 67%	Zero	100%	No	100%	67%	33%	No	100%	Crowding	33%	8.0 ± 2.8	Posterior cross-bite: 33%
**Treacher Collins Syndrome (*n* = 3)**	Convex: 100%	Zero	100%	No	100%	100%	No	50%	50%	Crowding	No	8.5 ± 1.0	Anterior open-bite: 100% Posterior cross-bite: 50%
**DiGeorge Syndrome (*n* = 3)**	Convex: 67% Concave: 33%	33%	100%	No	67%	67%	33%	33%	67%	Spacing	33%	1.5	Posterior cross-bite: 33%
**Prader–Willi Syndrome (*n* = 3)**	Straight: 33% Convex: 67%	No	67%	No	33%	33%	No	67%	33%	Spacing	No	4	Severe bruxism
**Ehlers–Danlos Syndrome (*n* = 2)**	Convex	100%	No	No	Yes	Yes	100%	No	100%	Crowding	100%	8	Anterior open bite
**Fetal Alcohol Syndrome (*n* = 1)**	Convex	No	Yes	No	Yes	Yes	No	Yes	No	Crowding	Yes	7	N/A
**Frontometaphyseal Dysplasia (*n* = 1)**	Straight	No	Yes	Yes	Yes	No	Yes	No	Yes	Spacing	No	10	Posterior open-bite
**Joubert Syndrome (*n* = 1)**	Convex	Yes	Yes	No	No	No	No	No	Yes	Spacing	No	4	Deviated nasal septum
**Neurofibromatosis Type I (*n* = 1)**	Straight	Yes	Yes	No	No	No	Yes	Yes	No	Crowding	No	1	Anterior cross-bite
**Ohdo Syndrome (*n* = 1)**	Convex	Yes	No	No	Yes	Yes	Yes	No	Yes	Crowding	No	3	Deep Overbite
**15q13.3 Microdeletion Syndrome (*n* = 1)**	Convex	Yes	Yes	No	Yes	Yes	No	No	No	Crowding	Yes	2	Deep overbite
**Dubowitz Syndrome** **(*n* = 1)**	Straight profile	Yes	Yes	No	No	No	Yes	No	Yes	Spacing	Yes	3	Deep overbite
**Cleft lip and palate** **(*n* = 1)**	Concave	Yes	Yes	Yes	No	No	No	Yes	No	Crowding	No	9	Anterior open-bite and cross-bite Posterior cross-bite
**Coffin–Siris Syndrome (*n* = 1)**	convex	No	Yes	No	Yes	Yes	No	Yes	No	Spacing	No	9	N/A

## Data Availability

Not applicable.

## Abbreviations

AASM	American Academy of Sleep Medicine
AHI	apnea–hypopnea index
BMI	body mass index
BMIP	body mass index percentile
DS	Down’s syndrome
ODI	oxygen desaturation index
OR	odds ratio
OSA	obstructive sleep apnea
PSG	polysomnography
SD	standard deviation

## References

[1] Caron C.J., Pluijmers B.I., Joosten K.F., Mathijssen I.M.J., Van Der Schroeff M.P., Dunaway D.J., Koudstaal M.J. (2015). Obstructive sleep apnoea in craniofacial microsomia: A systematic review. Int. J. Oral Maxillofac. Surg..

[2] Li H.-Y., Lee L.-A. (2009). Sleep-disordered breathing in children. Chang. Gung Med. J..

[3] Sheldon S.H., Ferber R., Kryger M.H. (2005). Principles and Practice of Pediatric Sleep Medicine.

[4] Bixler E.O., Vgontzas A.N., Lin H.M., Liao D., Calhoun S., Vela-Bueno A., Graff G. (2009). Sleep disordered breathing in children in a general population sample: Prevalence and risk factors. Sleep.

[5] Kheirandish-Gozal L., Gozal D. (2012). Sleep Disordered Breathing in Children: A Comprehensive Clinical Guide to Evaluation and Treatment.

[6] White S.C., Pharoah M.J. (2014). Oral Radiology: Principles and Interpretation.

[7] Lee R.W., Chan A.S., Grunstein R.R., Cistulli P.A. (2009). Craniofacial phenotyping in obstructive sleep apnea—A novel quantitative photographic approach. Sleep.

[8] Ayers E. (2017). Altered Craniofacial Morphology in Children with OSAS: A Clinical and Photographic Study. Ph.D. Thesis.

[9] Sutherland K., Weichard A.J., Davey M.J., Horne R.S., Cistulli P.A., Nixon G.M. (2020). Craniofacial photography and association with sleep-disordered breathing severity in children. Sleep Breath.

[10] McKusick V.A., McKusick V. (1998). Mendelian Inheritance in Man: A Catalog of Human Genes and Genetic Disorders.

[11] Shott S.R., Amin R., Chini B., Heubi C., Hotze S., Akers R. (2006). Obstructive sleep apnea: Should all children with Down syndrome be tested?. Arch. Otolaryngol. Head Neck Surg..

[12] Lal C., White D.R., Joseph J.E., van Bakergem K., LaRosa A. (2015). Sleep-disordered breathing in Down syndrome. Chest.

[13] Maris M., Verhulst S., Wojciechowski M., Van de Heyning P., Boudewyns A. (2016). Prevalence of obstructive sleep apnea in children with Down syndrome. Sleep.

[14] Hill C.M., Evans H.J., Elphick H., Farquhar M., Pickering R.M., Kingshott R., Gringras P. (2016). Prevalence and predictors of obstructive sleep apnoea in young children with Down syndrome. Sleep Med..

[15] Dyken M.E., Lin-Dyken D.C., Poulton S., Zimmerman M.B., Sedars E. (2003). Prospective polysomnographic analysis of obstructive sleep apnea in Down syndrome. Arch. Pediatr. Adolesc. Med..

[16] Simpson R., Oyekan A.A., Ehsan Z., Ingram D.G. (2018). Obstructive sleep apnea in patients with Down syndrome: Current perspectives. Nat. Sci. Sleep.

[17] Berenguer M., Tingaud-Sequeira A., Colovati M., Melaragno M.I., Bragagnolo S., Perez A., Rooryck C. (2017). A novel de novo mutation in MYT1, the unique OAVS gene identified so far. Eur. J. Hum. Genet..

[18] Baugh A., Wooten W., Chapman B., Drake A., Vaughn B. (2015). Sleep characteristics in Goldenhar syndrome. Int. J. Pediatr. Otorhinolaryngol..

[19] Christensen D., Van Naarden Braun K., Doernberg N.S., Maenner M.J., Arneson C.L., Durkin M.S., Yeargin-Allsopp M. (2014). Prevalence of cerebral palsy, co-occurring autism spectrum disorders, and motor functioning—Autism and Developmental Disabilities Monitoring Network, USA, 2008. Dev. Med. Child Neurol..

[20] Elsayed R.M., Hasanein B.M., Sayyah H.E., El-Auoty M.M., Tharwat N., Belal T.M. (2013). Sleep assessment of children with cerebral palsy: Using validated sleep questionnaire. Ann. Indian Acad. Neurol..

[21] Simard-Tremblay E., Constantin E., Gruber R., Brouillette R.T., Shevell M. (2011). Sleep in children with cerebral palsy: A review. J. Child Neurol..

[22] Anderson I.C.W., Sedaghat A.R., McGinley B.M., Redett R.J., Boss E.F., Ishman S.L. (2011). Prevalence and severity of obstructive sleep apnea and snoring in infants with Pierre Robin sequence. Cleft Palate-Craniofacial J..

[23] Daniel M., Bailey S., Walker K., Hensley R., Kol-Castro C., Badawi N., Waters K. (2013). Airway, feeding and growth in infants with Robin sequence and sleep apnoea. Int. J. Pediatr. Otorhinolaryngol..

[24] Sher A.E. (1992). Mechanisms of airway obstruction in Robin sequence: Implications for treatment. Cleft Palate-Craniofacial J..

[25] Kennedy W.P., Mudd P.A., Maguire M.A., Souders M.C., McDonald-McGinn D.M., Marcus C.L., Elden L.M. (2014). 22q11. 2 Deletion syndrome and obstructive sleep apnea. Int. J. Pediatr. Otorhinolaryngol..

[26] Johnston C., Taussig L., Koopmann C., Smith P., Bjelland J. (1981). Obstructive sleep apnea in Treacher-Collins syndrome. Cleft Palate J..

[27] Plomp R.G., van Lieshout M.J., Joosten K.F., Wolvius E.B., van der Schroeff M.P., Versnel S.L., Mathijssen I.M. (2016). Treacher Collins syndrome: A systematic review of evidence-based treatment and recommendations. Plast. Reconstr. Surg..

[28] Akre H., Øverland B., Åsten P., Skogedal N., Heimdal K. (2012). Obstructive sleep apnea in Treacher Collins syndrome. Eur. Arch. Oto-Rhino-Laryngol..

[29] Lin H.Y., Lin S.P., Lin C.C., Tsai L.P., Chen M.R., Chuang C.K., Huang C.Y. (2007). Polysomnographic characteristics in patients with Prader–Willi syndrome. Pediatr. Pulmonol..

[30] Al-Saleh S., Al-Naimi A., Hamilton J., Zweerink A., Iaboni A., Narang I. (2013). Longitudinal evaluation of sleep-disordered breathing in children with Prader-Willi Syndrome during 2 years of growth hormone therapy. J. Pediatr..

[31] Sedky K., Bennett D.S., Pumariega A. (2014). Prader Willi syndrome and obstructive sleep apnea: Co-occurrence in the pediatric population. J. Clin. Sleep Med..

[32] Zucconi M., Weber G., Castronovo V., Ferini-Strambi L., Russo F., Chiumello G., Smirne S. (1996). Sleep and upper airway obstruction in children with achondroplasia. J. Pediatr..

[33] Lee K.C.-H. (2015). Dentofacial Morphology in Children with Obstructive Sleep Apnea. Master’s Thesis.

[34] Shaw W., Richmond S., O’Brien K. (1995). The use of occlusal indices: A European perspective. Am. J. Orthod. Dentofac. Orthop..

[35] Kaditis A.G., Alvarez M.L.A., Boudewyns A., Alexopoulos E.I., Ersu R., Joosten K., Larramona H., Miano S., Narang I., Trang H. (2016). Obstructive sleep disordered breathing in 2-to 18-year-old children: Diagnosis and management. Eur. Respir. J..

[36] Flores-Mir C., Korayem M., Heo G., Witmans M., Major M.P., Major P.W. (2013). Craniofacial morphological characteristics in children with obstructive sleep apnea syndrome: A systematic review and meta-analysis. J. Am. Dent. Assoc..

[37] Katyal V., Pamula Y., Martin A.J., Daynes C.N., Kennedy J.D., Sampson W.J. (2013). Craniofacial and upper airway morphology in pediatric sleep-disordered breathing: Systematic review and meta-analysis. Am. J. Orthod. Dentofacial. Orthop..

[38] Bull M.J. (2011). Health supervision for children with Down syndrome. Pediatrics.

[39] Jheeta S., McGowan M., Hadjikoumi I. (2013). Is oximetry an effective screening tool for obstructive sleep apnoea in children with Down syndrome?. Arch. Dis. Child..

[40] Skotko B.G., Macklin E.A., Muselli M., Voelz L., McDonough M.E., Davidson E., Allareddy V., Jayaratne Y.S.N., Bruun R., Ching N. (2017). A predictive model for obstructive sleep apnea and Down syndrome. Am. J. Med. Genet. A..

[41] Ng C. (2018). Craniofacial Morphology in Children with Obesity or Down Syndrome with and without Obstructive Sleep Apnea. Master’s Thesis.

[42] Ørntoft M., Andersen I.G., Homøe P. (2020). Night-to-night variability in respiratory parameters in children and adolescents examined for obstructive sleep apnea. Int. J. Pediatr. Otorhinolaryngol..

